# Endoscopic mask innovation and protective measures changes during the coronavirus disease‐2019 pandemic: Experience from a Chinese hepato‐biliary‐pancreatic unit

**DOI:** 10.1111/den.13799

**Published:** 2020-09-18

**Authors:** Qing Tian, Xiaodong Yan, Rui Shi, Guijie Wang, Xiaomei Xu, Hongyan Wang, Qingqing Wang, Long Yang, Zirong Liu, Lanying Wang, Dhan Bahadur Shrestha, Yamin Zhang

**Affiliations:** ^1^ Department of Hepatobiliary Surgery Tianjin First Central Hospital Tianjin China; ^2^ First Central Clinic of Tianjin Medical University Tianjin China; ^3^ Department of Biotherapy Tianjin Medical University Cancer Institute and Hospital Tianjin China; ^4^ Tianjin Medical University Tianjin China

**Keywords:** COVID‐19, endoscopy, hepatobiliary, pandemic, SARS‐CoV‐2

## Abstract

Endoscopy is widely used as a clinical diagnosis and treatment method for certain hepatobiliary and pancreatic diseases. However, due to the distinctive epidemiological characteristics of severe acute respiratory syndrome coronavirus 2, the virus causing coronavirus disease‐2019 (COVID‐19), healthcare providers are exposed to the patient's respiratory and gastrointestinal fluids, rendering endoscopy a high risk for transmitting a nosocomial infection. This article introduces preventive measures for endoscopic treatment enacted in our medical center during COVID‐19, including the adjustment of indications, the application of endoscope protective equipment, the design and application of endoscopic masks and splash‐proof films, and novel recommendations for bedside endoscope pre‐sterilization.

## Introduction

In December 2019, coronavirus disease‐2019 (COVID‐19) broke out in Wuhan, Hubei Province, China, and quickly spread to other provinces and countries.^1^ According to epidemiological investigations, the main source of infection is patients with a severe acute respiratory syndrome coronavirus 2 (SARS‐CoV‐2) infection, which may also include asymptomatic infection.^2^, ^3^ Droplet transmission and close contact transmission are the main modes of transmitting SARS‐CoV‐2, but there is a possibility of aerosol transmission in a relatively closed environment, due to long‐term exposure to high concentrations of aerosol. The isolation of SARS‐CoV‐2 in feces and urine suggests that the virus may be spread by aerosols and close contact transmission caused by fecal and urine pollution in the environment.^4^, ^5^

For some hepatobiliary and pancreatic diseases, conservative treatment often cannot alleviate the patient's symptoms. Endoscopy is widely used as a clinical diagnosis and treatment method. During the COVID‐19 pandemic, due to the distinctive epidemiological characteristics of SARS‐CoV‐2, endoscopy poses a high risk of nosocomial infection, since healthcare providers are exposed to the patient's respiratory and gastrointestinal fluids.^6^ Therefore, to minimize infections among patients and medical personnel, identifying an orderly and safe method for emergency endoscopy treatments has become an urgent concern.

This article introduces the COVID‐19 prevention measures used in our medical center (Department of Hepatobiliary Surgery, Tianjin First Central Hospital). Building on the prevention and treatment experience of other countries or centers,^7^, ^8^, ^9^, ^10^ we offer several reasonable suggestions for conducting endoscopies during the pandemic, applying endoscope protection articles, and endoscope reprocessing.

## Procedure

### Patient risk classification and treatment principles

Based on their possibility of having COVID‐19, our patients are categorized as high, medium, or low risk. Patients with different risk levels are classified and treated in different ways (Table 1). The specific evaluation criteria are as follows^11^, ^12^: (i) any symptoms caused by SARS‐CoV‐2 infection, including but not limited to fever, cough, dyspnea, and gastrointestinal symptoms; (ii) any exposure to patients confirmed or suspected to have COVID‐19 in the past 14 days; (iii) travel history in countries or regions with a high incidence of COVID‐19 in the past 14 days; and (iv) travel history and health condition of family members.

**Table 1:** Risk level classification and treatment principles

Risk level	Definition	Disposal principles
High	Patients who were diagnosed as suspected cases due to the presenting symptoms at the fever clinic	Isolate the hot clinic; start the sampling process Severe patients are treated in the hot clinic, if necessary; our physician will conduct the consultation Refer to the designated hospital as soon as possible after the diagnosis Strictly control the hospitalization of patients with negative nucleic acid tests. Indicate and arrange admission to the isolation room Complete the diagnosis and treatment process, and let the patient leave the hospital as soon as possible after meeting the discharge criteria
Medium	Patients who were referred to the fever clinic due to the presenting symptoms and were excluded from the list of suspected cases	After ruling out the suspicion, carry out diagnosis and treatment in accordance with the principle of admission and treatment, and closely observe the body temperature, respiratory symptoms, and disease changes After a discussion with the prevention and control team, in principle, patients who must undergo endoscopic treatment are admitted in a single room, and they can be admitted to the general ward while ensuring a sufficient isolation period (at least 7 days) Strictly implement the hospital's standards for sensory control and reinforce the protection of patients and medical staff When the diagnosis and treatment process is completed, and let the patient leave the hospital as soon as possible in accordance with the discharge criteria
Low	Ordinary patients with no epidemiological history and related symptoms	The consultation physicians in our department are responsible for screening and evaluation. After a discussion with the prevention and control team, patients who must undergo endoscopic treatment can be admitted to the hospital for treatment In principle, patients are admitted in separate single rooms for treatment, and they can be admitted to ordinary wards after undergoing a sufficient isolation period (at least 7 days) Under the premise of strict implementation of the hospital's standard of sensory control for diagnosis and treatment, carry out diagnosis and treatment in accordance with standard procedures Observe the changes in the condition closely and standardize the protection of patients and medical staff

### Indications for hepatobiliary and pancreatic diseases using endoscopy for diagnosis and treatment

1. Common bile duct stones with acute cholangitis.
2. Malignant obstructive jaundice.
3. Adverse biliary events after liver transplantation, including biliary anastomosis stenosis, bile duct stones, bile leakage, and continuous deterioration of liver function.
4. Biliary pancreatitis.

### Training and protection of medical personnel

1. Set up a pandemic prevention and control group including surgical directors, endoscopic surgeons, and endoscopic nurses.
2. Carry out training on COVID‐19 and personal protection through a mobile phone app, a website, and a video presentation.
3. Establish a permanent and experienced endoscopic treatment team to manage outpatient and emergency patients with related diseases.
4. Develop a detailed list of personal protective equipment (PPE), including disposable protective clothing, N95 masks, medical masks, and face shields, to ensure that medical personnel have sufficient PPE to protect them from viruses.
5. Assign special personnel to conduct daily personal health surveys for all medical personnel in the department, including temperature, activities outside the hospital, etc.
6. All medical personnel who (i) have fever or respiratory symptoms, (ii) had contact with suspected or confirmed COVID‐19 patients, (iii) live in or had contact with individuals residing in areas where the disease is prevalent, or (iv) recently returned from a high‐pandemic area or country should undergo self‐isolation for 14 days. If necessary, a nucleic acid antibody staining test and related tests and examinations shall be carried out.
7. In the staff restroom or dining room, the separation distance should be increased to at least 1 m, to avoid cross‐infection caused by face‐to‐face contact.
8. Develop good hand hygiene habits and reduce the possibility of contact transmission.
9. Ensure sufficient rest time for medical personnel, to prevent low immune function and increased risk of virus infection due to overwork.

### Preoperative diagnosis and treatment

1. Open an Internet telemedicine system and telephone consultation service, arranging for doctors to provide consultation service to patients, in order to reduce patients' visit time, frequency, and process, thereby reducing the risk of hospital infection.
2. In order to shorten waiting time, consider adopting an outpatient appointment mode. An isolation waiting area should be set up outside the diagnosis room. The distance between each patient should be at least 1 m, and a maximum of one accompanying individual is allowed.^13^, ^14^ All patients and their accompanying individuals should wear medical masks and undergo epidemiological investigation.
3. Patients who have a fever and respiratory or gastrointestinal symptoms should participate in the COVID‐19 epidemiology survey at the fever clinic, and a routine blood test, C‐reactive protein (CRP) test, procalcitonin (PCT) test, and chest computed tomography (CT) examination should also be performed. A reverse transcription‐polymerase chain reaction (RT‐PCR) for SARS‐CoV‐2 should be carried out in suspected patients to eliminate the possibility of infection. The confirmed patients should immediately be prepared for admission to a separate isolation ward for treatment and then transferred to the COVID‐19 designated hospital in their area as soon as possible for centralized treatment. Isolation and medical observation should be implemented on the patients' close contacts. Those suspected of having COVID‐19 should be isolated in a separate isolation ward for diagnosis and treatment with a permanently designated medical team, and a repeat RT‐PCR should be performed to rule out COVID‐19 diagnosis.
4. For patients who do not have COVID‐19, the diagnosis and treatment of primary diseases can continue in the outpatient or emergency consulting room. Examination methods with higher accuracy should be selected. Patients with acute cholangitis, biliary pancreatitis, and complications after liver transplantation can be examined through an abdominal CT or magnetic resonance cholangiopancreatography (MRCP). Patients with malignant tumors can be examined using an abdominal enhanced CT. After the establishment of a diagnosis and a discussion by the prevention and control group, the patients who meet the indications for endoscopy treatment should be admitted in separate wards for further diagnosis and treatment. The isolation ward should be set up in a place with less foot traffic.
5. Follow‐up with all patients for 14 days, to avoid the possibility of hospital cross‐infection caused by asymptomatic infection.

### Intraoperative management

**Figure 1:**
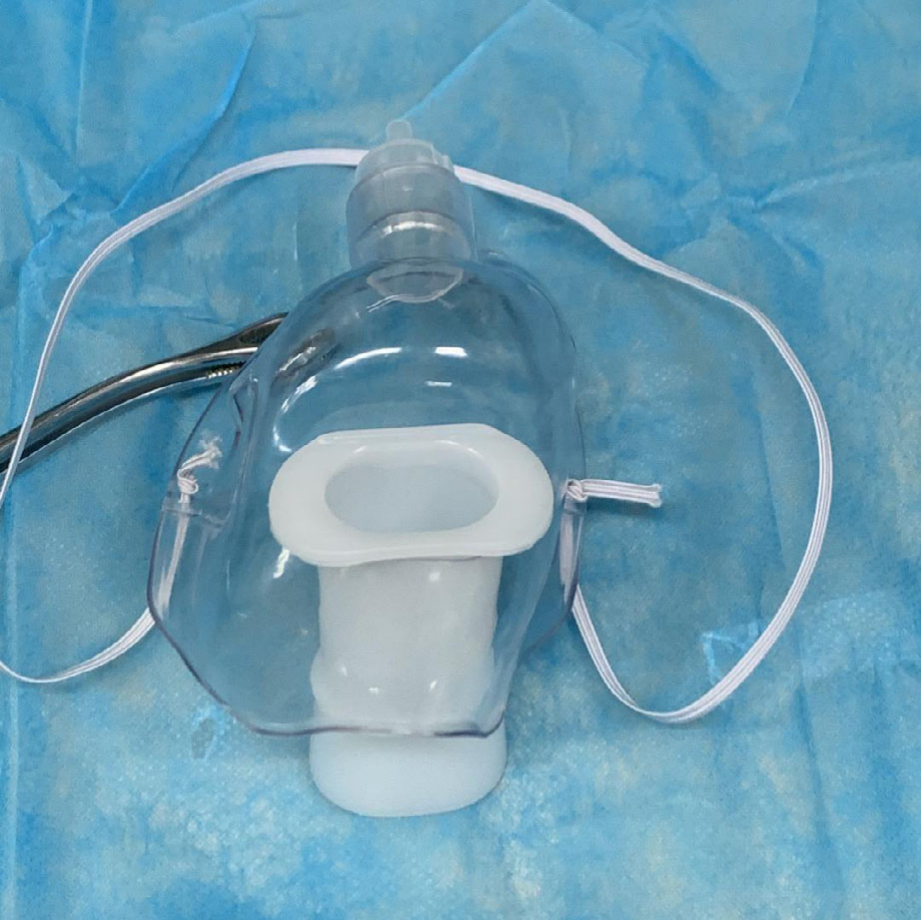
The duodenoscopy protective mask.

**Figure 2:**
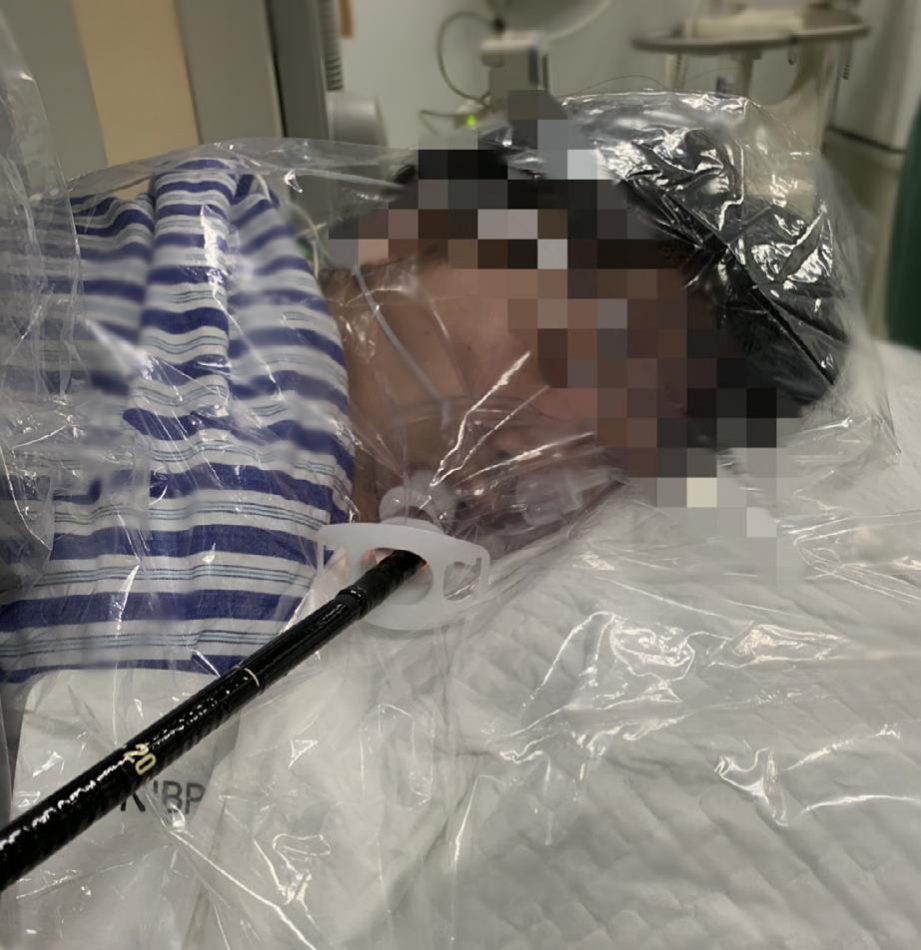
A patient with the duodenoscopy protective mask and the anti‐splashing membrane.

1. All patients should go to the endoscopy center from the designated channel for diagnosis and treatment. Patients should wear medical masks all the time, except during operations.
2. Endoscopy should be carried out in a fixed endoscopy room with the air conditioning and ventilation equipment closed, or in a negative‐pressure operating room. In addition, our center recommends that only an experienced endoscopist, an instrument nurse, and if necessary an anesthesiologist be kept in the endoscopic unit^15^.
3. During the treatment process, disposable treatment towels and bed sheets should be selected as much as possible, to prevent contamination caused by gastric juice splashing during the operation and cross‐infection among patients.
4. Our center recommends that patients be given no anesthesia or intravenous anesthesia during endoscopic procedures, to reduce the choking caused by intubation, which can cause droplets or aerosol transmission.
5. Medical personnel in the endoscopy clinic shall wear PPE, including medical masks/N95 masks, goggles/face shields, disposable waterproof surgical gowns, medical gloves, headgear, boot covers, etc. For patients with COVID‐19, medical personnel should wear N95 masks, goggles/face shields, disposable protective clothing, double gloves, headgear, and boot covers.
6. Our center reconstructed a simple duodenoscopy protective mask using a duodenoscopy bite block and a respiratory mask (Fig. 1). Use sterile scissors to cut an appropriate opening in front of the respiratory mask and place the bite block into the respiratory mask. The patient bites it and wears the mask before the operation. An oxygen catheter can be connected to the upper end of the mask to give oxygen to the patient during endoscopic operations. At the same time, the patient's body surface is covered with a disposable, transparent, waterproof plastic film containing operation holes. The duodenoscope is inserted into the patient's body through the operation hole and the bite block for treatment (Fig. 2). This operation can further cut off the transmission path of the virus, and relevant experiments have confirmed that the use of splash film can effectively reduce the spray range^16^. It is important that these items are disposable and easily accessible. This can avoid cross‐infection between patients and reduce indoor aerosol dissemination range, greatly improving the safety factor and utilization rate of the endoscope unit.
7. After treatment, our center also improved bedside pre‐processing. The enzyme cleaning agent we used, which had no sterilization or disinfection effect, was changed to weak alkaline peracetic acid. When the endoscope is removed from the patient's mouth, wipe the endoscope with sterile gauze moistened with weak alkaline peracetic acid disinfectant (2300 mg/L). Put the lens into a sealed plastic bag filled with disinfectant, start the suction function, and suck the disinfectant until it flows into the suction tube. Separate the endoscope from the host, put it in a disposable waterproof bag, put the waterproof bag in a double‐layer yellow plastic bag, and strap it. After putting the plastic bag in the article transfer box and closing it, transfer it to the disinfection center for reprocessing according to a fixed transfer route^17^.
8. After diagnosis and treatment, the endoscopy unit should undergo disinfection at the following rates. (i) In a fixed endoscopy operation unit, use ultraviolet disinfection twice a day for at least 30 min, as well as automatic air disinfection and machine disinfection. (ii) After each operation, use 1000 mg/L of chlorine disinfectant to wipe all the tabletops, articles, and floors in the diagnosis and treatment room, then clean with water after 30 min. (iii) After each operation, use 75% ethanol to wipe and disinfect the endoscope host, monitor, operating platform, and other equipment.

### Postoperative management

1. After endoscopic treatment, transfer the patient back to the ward through a fixed route and minimize going out as much as possible. Arrange a fixed escort to care for the patient.
2. For suspected or confirmed COVID‐19 patients, our center recommends using double‐layer medical garbage bags to seal the patient's excreta, sending the bags to a medical waste treatment center for disinfection, and then discharging them again.
3. Patients should develop good hand hygiene habits to prevent fecal–oral virus transmission.
4. Our patients usually have a fever, cholangitis, and other symptoms before surgery. Therefore, our center recommends using a biliary stent instead of a nasobiliary duct for biliary drainage as much as possible, to avoid the risk of hospital infection caused by the external drainage of bile. After surgery, anti‐inflammatory treatment with broad‐spectrum antibiotics is necessary for patients at risk of biliary infection. Based on the results of imaging and laboratory examinations, perform differential diagnosis on fever patients again.
5. Patients with hepatobiliary and pancreatic diseases often have different degrees of eating disorders before surgery, resulting in malnutrition. The Nutritional Risk Screening Scale 2002 (NRS‐2002)^18^ can be used for scoring. When NRS‐2002 ≥3 points, there is a nutritional risk. Provide nutritional support treatment at the same time as conventional treatment. For patients with contraindications to feeding, parenteral nutrition is supplemented, but for those without contraindications, start enteral nutrition as soon as possible. This protects the functions of important organs such as the liver and kidney, improves the nutritional status and immune function of patients, and prevents SARS‐CoV‐2 infection.
6. Because of the double burden of gastrointestinal disease and COVID‐19, patients often demonstrate negative emotions such as irritability and anxiety^19^. Therefore, psychological counseling is an essential component of comprehensive treatment for postoperative patients. According to each patient's condition, individual psychological intervention should alleviate the patients' concerns and enhance their confidence by giving appropriate suggestions and psychological guidance^20^.
7. Patients who meet the discharge criteria should be discharged as soon as possible, to avoid hospital infection caused by long‐term exposure to the hospital environment. At the same time, follow‐up with the patients and their accompanying individuals for 14 days, to rule out the possibility of asymptomatic infection.
8. The discharged patients can avail remote follow‐up consultation services with competent doctors through an Internet telemedicine system, a mobile app, and other methods, to reduce the number of visits and avoid the risk of cross‐infection in the hospital.

## Discussion

The potent virulence of SARS‐CoV‐2 has resulted in a worldwide pandemic. The course of hepatobiliary and pancreatic diseases is not only rapid but also critical, often requiring urgent treatment. Each center should establish a corresponding endoscopic diagnosis and treatment process according to the prevalent medical conditions, equipment availability, policy requirements, and the latest research results on COVID‐19. With the aim of fully protecting the medical staff from cross‐infection, provide necessary medical services for emergency patients and avoid the possibility of cross‐infection in the hospital. At the same time, the implementation of psychological counseling for medical staff and patients should be considered to prevent the psychological impacts of COVID‐19.

## Conflict of Interest

Authors declare no Conflict of Interests for this article.

## Funding Information

This work was supported by funding from the Tianjin Science and Technology Plan Project [19ZXDBSY00010], Key Projects of Tianjin Health Industry [16KG108], and Tianjin First Central Hospital Spring Bud Project [2019CL01].
